# Oral Quercetin Supplementation Enhances Adiponectin Receptor Transcript Expression in Polycystic Ovary Syndrome Patients: A Randomized Placebo-Controlled Double-Blind Clinical Trial

**DOI:** 10.22074/cellj.2018.4577

**Published:** 2017-11-04

**Authors:** Neda Rezvan, Ashraf Moini, Sattar Gorgani-Firuzjaee, Mohammad Javad Hosseinzadeh-Attar

**Affiliations:** 1 Department of Clinical Nutrition, School of Nutritional Sciences and Dietetics, International Campus, Tehran University of Medical Sciences, Tehran, Iran; 2 Department of Obstetrics and Gynecology, Faculty of Medicine, Tehran University of Medical Sciences, Tehran, Iran; 3 Department of Obstetrics and Gynecology, Arash Women’s Hospital, Tehran University of Medical Sciences, Tehran, Iran; 4 Department of Medical Laboratory Sciences, School of Allied Health Medicine, AJA University of Medical Sciences, Tehran, Iran

**Keywords:** Adiponectin Receptors, Polycystic Ovary Syndrome, Quercetin

## Abstract

**Objective:**

Polycystic ovary syndrome (PCOS), an ovarian-pituitary axis androgen disorder, is a common endocrine disease in women. Obesity-induced androgenesis and imbalance of adipokine secretion may lead to some metabolic features of PCOS. The beneficial effects of polyphenolic compounds such as quercetin have been reported, however, the underlying molecular mechanism is not entirely understood. In the present study, we investigated the effect of quercetin supplementation on the expression of adiponectin receptors at the transcript level in peripheral blood mononuclear cells (PBMC) samples of PCOS patients.

**Materials and Methods:**

In this randomized clinical trial, 84 PCOS subjects were randomly assigned to two groups; the treatment group received 1 g quercetin (two 500 mg capsules) daily for 12 weeks and the control group received placebo. To examine the effect of quercetin supplementation on PCOS patients in addition to biochemical and anthropometric assessments, the expression of *ADIPOR1* and *ADIPOR2* at the transcript level and AMPK level were determined by quantitative reverse transcription-polymerase chain reaction (RT-qPCR) and ELISA assays respectively.

**Results:**

Oral quercetin supplementation significantly increased *ADIPOR1* and *ADIPOR2* transcript expression by 1.32- and 1.46-fold respecetively (P<0.01). In addition, quercetin supplementation enhanced AMPK level by 12.3% compared with the control group (P<0.05).

**Conclusion:**

Oral quercetin supplementation improves the metabolic features of PCOS patients by upregulating the expression of adiponectin receptors and AMPK (Registration Number: IRCT2013112515536N1).

## Introduction

Polycystic ovary syndrome (PCOS) is a multifactorial metabolic disorder with an unknown underlying molecular mechanism. Hyperinsulinemia, a consequence of insulin resistance, is a culprit of PCOS pathogenesis (1, 2). In addition, obesity substantially exacerbates this detrimental condition where reproductive-age obese women with PCOS are very prone to glucose intolerance (3). Previous studies have reported that an excessive visceral fat distribution and an imbalanced adipokine profile are not only markers of insulin resistance, but they also can trigger PCOS development via activation of the pituitary-ovary axis (4, 5). Adipokines are peptide hormones mostly derived from the adipose tissue (6). Several peptide hormones and adipokines (such as adiponectin, omentin and ghrelin) are present abnormally in women with PCOS (7). It is thought that leptin and adiponectin enhance the release of growth hormones (8), the follicle stimulating hormone (FSH), the luteinizing hormone (LH) (9) and the thyroid stimulating hormone (TSH) by activating their pituitary receptors (6). Also, some adipokines directly affect ovarian steroidogenesis (1, 3) while others (e.g. adiponectin and omentin) play beneficial roles in glucose homeostasis by increasing insulin-stimulated glucose uptake via Akt phosphorylation (10, 11). Moreover, they reduce cardiovascular risk through amelioration of inflammation (6, 12). The impaired adiponectin-mediated insulin sensitivity is observed in obese individuals including PCOS patients (13, 14). Moreover, previous studies have demonstrated low levels of adiponectin in PCOS subjects even in the absence of adiposity (15).

Adiponectin signaling is controlled by its receptors, *ADIPOR1* and *ADIPOR2*, which regulate glucose and fatty acid metabolism partly via the activation of AMP-activated protein kinase (AMPK) (2). *ADIPOR1* and *ADIPOR2* belong to the G protein-coupled receptors family. However, in contrast to common G protein-coupled receptors, their N-terminus is internal while the C-terminus is external (16). It has been shown that adiponectin receptors are upregulated at the transcript and protein levels in adipocytes of PCOS women but not in their peripheral blood mononuclear cells (PBMC) (17). Quercetin (3,3ʹ,4ʹ,^5^,^7^- pentahydroxyflavone), a flavonoid with antioxidant and anti-inflammatory properties, is found in food such as apples and onions, and drinks such as tea and red wine (18). A large body of evidence has shown its beneficial effects on metabolic disorders such as obesity, insulin resistance and cardiovascular disease (19). Previous studies on animal models have illustrated that administration of this flavonoid ameliorates dyslipidemia, hypertension, hyperinsulinemia and tumor necrosis factoralpha (TNF-α) production (20). Also, it has been shown that quercetin supplementation in high fat diet-induced obese mice resulted in lower levels of glucose, insulin, triglycerides and cholesterol but increased the secretion of adiponectin. This finding confirmed the benefecial effects of quercitin administration on methabolic features of obesity (21). Importantly, the bioavailability of oral administration of quercetin was observed in healthy subjects (22). In humans, quercetin induces nitric oxide release and reduces the production of free radicals, which in turn improve blood pressure and anti-atherosclerosis capacity (23, 24).

To the best of our knowledge, these beneficial effects are most likely exerted through the adipokine-mediated insulin signaling pathway (25, 26). However, no study has directly examined the effect of quercetin supplementation on the expression of adiponectin receptor genes. We therefore investigated the effects of quercetin supplementation on adiponectin receptor expression at the transcript level in PBMC and potential adiponectin-mediated expression changes of AMPK in PCOS patients.

## Materials and Methods

This clinical trial was a randomized placebo-controlled double-blind trial with a superiority single-arm design. Women with PCOS were randomly divided into two equal parallel groups, receiving either quercetin supplement or placebo. The study protocol was approved by the Ethical Committee of Tehran University of Medical Sciences. This study was registered in the Iranian Registry of Clinical Trials (IRCT2013112515536N1). Eighty-four PCOS women diagnosed according to the 2003 Rotterdam criteria (27) were voluntarily recruited at Arash Hospital (Tehran, Iran) from January 2014 to January 2015. All women were 20-40 years of age with body mass index (BMI) ranging from 25 to 40 kg/m^2^. Patients with no history of simultaneous endocrine or metabolic diseases (e.g. hypo- or hyperthyroidism, androgensecreting tumors, diabetes mellitus, adrenal hyperplasia and Cushing’s syndrome) were enrolled only. Furthermore, patients who were prescribed with interfering medications (e.g. metformin and contraceptive, antihypertensive, antiglycemic, anti-hyperlipidemia or anti-inflammatory drugs) were excluded. All the recruited patients were informed about the purpose of the study and completed a written informed consent. Patients were free to discontinue the trial at any time during the study.

This clinical trial consisted of a 12-week treatment with quercetin or placebo (28, 29). The quercetin group (n=42) was given one gram daily (Jarrow, USA) in the form of two 500-mg capsules after each main meal (breakfast and lunch). Song et al. (30) have reported that the most effective dose of quercetin on glycemic status is 60 mg/ kg, approximately equivalent to 4 g for an adult human, however, importantly, quercetin supplementations have been shown to be beneficial in humans even with lower doses (e.g., 500 mg/daily and 1000 mg/daily) (30-32). The Placebo group (n=42) was given two starch-containing capsules for 12 weeks. Placebo capsules were in the same shape as quercetin capsules and were produced at the School of Pharmacy of Tehran University of Medical Sciences. We monitored the compliance of the volunteers by counting the returned capsules every two weeks. Participants were asked to maintain their diet and physical activity during the study.

### Anthropometric and biochemical analysis

Body weight was measured by a scale (Seca, Germany) with patients wearing no shoes and having light clothing. Height was measured by a metric mounted tape. BMI was then calculated as weight over squared height (kg/m^2^). Waist and hip circumference (WC and HiC) were determined using a soft metric tape in standing position. It is worth noting that WC was measured at the narrowest circumference between the costal margin and the iliac crest, and HiC at the widest circumference between the waists and tights. Waist to hip ratio (WHR) was then calculated as WC in centimeters divided by HiC in centimeters. Systolic and diastolic blood pressures (SBP and DBP respectively) were checked by a sphygmomanometer (Rester) in a sitting position after 5-10 minutes rest. Blood samples (12 ml) were collected after 12 hours of fasting between 7 and 9 am. Blood samples were centrifuged at 4000 rpm for 5 minutes, and sera were then stored at -80˚C until further analysis. Biochemical parameters such as fasting blood sugar and lipid profile [triglycerides (TG), total cholesterol, high-density lipoproteins-cholesterol (HDL-C), and low-density lipoproteins- cholesterol (LDL-C)] were measured by Pars Azmon enzymatic methods and analyzed on a Hitachi 717 autoanalyser.

### Peripheral blood mononuclear cells isolation

PBMC were isolated from 7 ml heparinized whole blood by using a Ficoll-Hypaque density gradient centrifugation. The PBMC were washed twice with phosphate-buffered saline (PBS) and stored at -70˚C until RNA extraction.

### RNA extraction and first strand cDNA synthesis

Total RNA of collected PBMCs was isolated using the total RNA Extraction RNeasy Mini Kit (Qiagen, Germany). Its quantity and quality were examined by Nanodrop and agarose gel electrophoresis respectively. To eliminate the effect of different number of isolated PBMC from patients, 500 ng of total RNA was reverse transcribed to cDNA with the M-MuLV reverse transcriptase and random hexamer primers (miScript II RT Kit, Qiagen) in 2 steps. Initially, a polyadenylation reaction was carried out at 37˚C for 30 minutes and then, the reverse transcription step was conducted.

### Quantitative real-time polymerase chain reaction

Quantitative real time-PCR was performed using the SYBR Green Premix EX Taq II (Takara Biotechnology, LTD, Dalian, Japan). Gene expression levels were quantified using specific primers for *ADIPOR1, ADIPOR2* and *β-actin* as an internal control (Table 1). The levels of target gene transcripts were normalized relative to those of β actin (delta CT). The amplification protocol was an initial activation step for 10 seconds at 95˚C followed by 40 cycles of 5 seconds at 95˚C for denaturation and 20 seconds at 60˚C for annealing/ extension.

**Table 1:** Primers sequences used in this study


Primer	Primer sequencing (5ˊ-3ˊ)

*ADIPOR1*	F: CTTCTACTGCTCCCCACAGC
	R: GACAAAGCCCTCAGCGATAG
*ADIPOR2*	F: CATGTCCCCTCTCTTACAAG
	R: ATGTGTCCAAATGTTGCCTG
*β-actin*	F: ATAGCACAGCCTGGATAGCAACGTAC
	R: CACCTTCTACAATGAGCTGCGTGTG


### Serum p-AMPK measurements

Serum p-AMPK levels were measured by human phosphorylated adenosine monophosphate activated protein kinase (AMPK) ELISA kit (Bioassay Technology Laboratory, China).

### Statistical analyses

Quantitative variables were presented as mean (± SD or SE where appropriate). The independent t test was used to compare the mean of quantitative variables between two groups. We used the analysis of covariance (ANCOVA) to compare the mean of post-intervention continuous outcomes between two groups by adjusting for preintervention results. Statistical analyses were undertaken in SPSS 20.0 (IBM SPSS Statistics for Windows, Version 20.0. Armonk, NY: IBM Corp). P<0.05 were considered statistically significant.

## Results

General characteristics of the two groups showed that all anthropometric features were similar in the two studied groups except for DBP (Table 2). The comparison of crude and adjusted values of post-intervention quercetin and placebo groups were not significantly different with respect to BMI, WHR and weight (Table 3).

After controlling for relevant confounding factors including age, DBP and sex hormone binding globulin LH (SHBG) and considering baseline values of each variable as covariates, the metabolic profile showed a significant difference between the two groups. Moreover, FBS and insulin levels, as well as calculated HOMA-IR as an index of insulin resistance, were significantly lower in the quercetin group compared with the placebo group (Table 4).

### Quercetin supplementation induced adiponectin receptor expression

**Fig.1:**
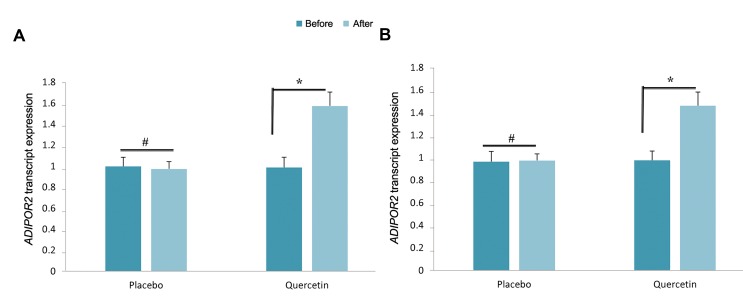
Effect of quercetin supplementation on adiponectin receptor transcript expression. A. *ADIPOR1* transcript expression and B. *ADIPOR2* transcript expression. All 42 samples in each group were run in duplicate. *; P<0.01 and #; P>0.05.

To investigate the molecular mechanism of the effect of quercetin supplementation on crosstalk between adipose tissue and reproductive system, we targeted *ADIPOR1* and *ADIPOR2* transcript expression in isolated PBMC of PCOS patients. Quercetin supplementation significantly upregulated both *ADIPOR1, ADIPOR2* transcript expression (1.32- and 1.46-fold respectively, P<0.01, Fig .1).

**Table 2:** Clinical and biochemical characteristics of the study participants in the quercetin and placebo groups


Baseline Characteristic	Quercetin n=42	Placebo n=40	P value^*^

Age (Y)	29.45 (4.09)	30.00 (5.44)	0.607
Weight) kg)	76.46 (11.89)	74.89 (11.74)	0.550
BMI (kg/m^2^)	29.32 (3.71)	28.61 (4.06)	0.411
WHR(cm)	0.83 (0.02)	0.83 (0.02)	0.233
SBP(mmHg)	12.63 (0.66)	12.56 (0.67)	0.644
DBP(mmHg)	8.79 (0.72)	8.43 (0.64)	0.018
FBS (mg/dL)	91.57 (6.74)	89.83 (6.36)	0.231
Insulin (μIU/mL)	10.09 (2.78)	9.91 (2.56)	0.754
HOMA-IR	2.28 (0.72)	2.20 (0.68)	0.604
SHBG ( nmol/l)	41.04 (17.73)	35.62 (13.04)	0.120
LH(mIU/ml)	8.40 (3.03)	8.67 (2.07)	0.644
Testosterone	0.78 (0.16)	0.78 (0.15)	0.941
Adiponectin (Total) (ng/mL)	9.58 (1.80)	10.05 (1.24)	0.163
Adiponectin (HMW)	5.12 (1.01)	5.30 (0.91)	0.421
All values are mean (SD). BMI; Body mass index, WHR; Waist to hip ratio, SBP; Systolic blood pressure, DBP; Diastolic blood pressure, FBS; Fasting blood sugar, LH; Luteinizing hormone, SHBG; Sex hormone binding globulin LH, HOMA-IR; Homeostasis model assessment-insulin resistance, HMW; High molecular weight, and *; Significances are based on independent t test.			


All values are mean (SD). BMI; Body mass index, WHR; Waist to hip ratio, SBP; Systolic blood pressure, DBP; Diastolic blood pressure, FBS; Fasting blood sugar, LH; Luteinizing hormone, SHBG; Sex hormone binding globulin LH, HOMA-IR; Homeostasis model assessment-insulin resistance, HMW; High molecular weight, and *; Significances are based on independent t test.

**Table 3:** Post-intervention anthropometric characteristics of women with PCOS randomly allocated into the placebo and treatment (quercetin) groups


Characteristic	Quercetin n=42		Placebo n=40		Crude P value	Adjusted P value
	Crude	Adjusted	Crude	Adjusted		

Weight (kg)	76.13 (1.85)	75.40 (0.72)	74.82 (1.85)	75.59 (0.70)	0.618	0.085
BMI	29.19 (0.58)	28.86 (0.03)	28.59 (0.64)	28.93 (0.03)	0.485	0.054
WHR	0.83 (0.01)	0.83 (0.00)	0.83 (0.01)	0.83 (0.00)	0.584	0.107


All values are mean (SE). Crude comparisons are based on independent t test and adjusted comparisons are based on ANCOVA with variables age, DBP, SHBG and considering baseline value of each variable as covariates. PCOS; Polycystic ovary syndrome, BMI; Body mass index, WHR; Waist to hip ratio, DBP; Diastolic blood pressure, and SHBG; Sex hormone binding globulin.

**Table 4:** Post-intervention metabolic profile of women with PCOS randomly allocated into the placebo and treatment (quercetin) groups


Characteristic	Quercetin n=42		Placebo n=40		Crude P value	Adjusted P value
	Crude	Adjusted	Crude	Adjusted		

Insulin (μIU/mL)	8.45 (0.34)	8.35 (0.15)	9.81 (0.41)	9.91 (0.15)	0.013^*^	<0.001^*^
FBS (mg/dl)	90.36 (1.03)	89.58 (0.23)	89.45 (0.98)	90.27 (0.23)	0.526	0.045^*^
HOMA-IR	1.88 (0.09)	1.84 (0.03)	2.17 (0.11)	2.21 (0.04)	0.039	<0.001^*^
All values are mean (SE). Crude Significances are based on independent t test and adjusted significances are based on ANCOVA with variables age, BMI, DBP, SHBG and considering baseline value of each variable as covariates. PCOS; Polycystic ovary syndrome, FBS; Fasting blood sugar, HOMA-IR; Homeostasis model assessment-insulin resistance, and ^*^; Statistically significant (P<0.05).						


All values are mean (SE). Crude Significances are based on independent t test and adjusted significances are based on ANCOVA with variables age, BMI, DBP, SHBG and considering baseline value of each variable as covariates. PCOS; Polycystic ovary syndrome, FBS; Fasting blood sugar, HOMA-IR; Homeostasis model assessment-insulin resistance, and *; Statistically zsignificant (P<0.05).

### Quercetin supplementation induced AMPK

**Fig.2:**
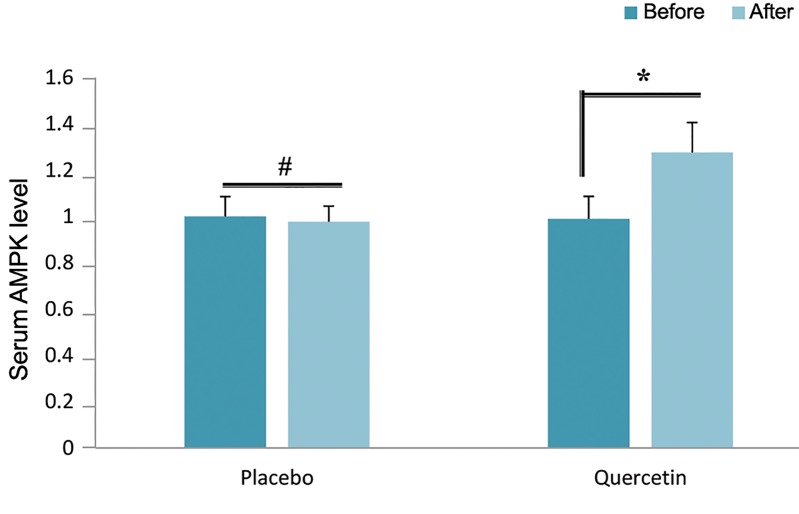
Effect of quercetin supplementation on serum AMPK level. *; P<0.01 and #; P>0.05.

To further investigate the effect of the adiponectin receptor signaling pathway, we analyzed the expression of a downstream molecular target, AMPK. The results showed that quercetin supplementation enhances the AMPK level by 12.3% compared with the control group (P<0.05) (Fig .2).

## Discussion

This study provides direct evidence for a novel link between quercetin, a compound of alternative medicine, and metabolic features of PCOS via adiponectin receptors and AMPK. We demonstrated that quercetin supplementation upregulated adiponectin receptors (*ADIPOR1* and *ADIPOR2*) and AMPK at the transcript and protein level respectively in PCOS patients. Adiponectin, the prominent human adipokine, is usually reduced in obese PCOS subjects (8). We have previously shown that quercetin supplementation enhances adiponectin levels in PCOS women (33). According to in vitro and in vivo studies on other polyphenols such as resveratrol it is established that adiponectin is increased by these supplements (34).

Induction of adiponectin receptors in PBMC of PCOS women in the presence of quercetin suggests that quercetin supplementation enhances adiponectin signal transduction, which is responsible for the beneficial hypolipidemic effects of quercetin supplementation in PCOS patients. Consistent with our finding, previous studies have found that the expression of adiponectin receptors is elevated in adipose tissue of PCOS women, thus enhancing adiponectin signaling in adipose tissue (17). In addition, Zhang et al. (35) showed that induction of *ADIPOR1* and *ADIPOR2* in PCOS patients leads to the early development of the embryo. More importantly, Tan et al. (36) demonstrated that elevation of adiponectin receptors at the transcript level is associated with the in vivo clearance of triglycerides in postprandial states. Also, Comim et al. (37) reported that reduction in serum adiponectin levels and downregulation of its receptors causes defects in its signaling. Interestingly, the lower level of adiponectin and adiponectin receptors in theca cells significantly contributes to the exacerbation of hyperandrogenism in overweight or obese women with PCOS. Furthermore, they demonstrated that other adipokines, such as leptin and visfatin can slightly enhance the basal androgen secretion in bovine theca cells via unknown mechanisms. Leiherer et al. (38) reported that quercetin also altered expression of some adipokines including Angptl4, adipsin, irisin and PAI-1 in addition to glucose catabolism genes, ENO2, PFKP and PFKFB4. They suggested that quercetin supplementation exerts its beneficial effects not only by its antioxidant capacity but also by targeting metabolic pathways and adipokine expression.

Altogether, reduction of adiponectin and its receptors is a key mechanism which links metabolic and reproductive dysfunctions in women with PCOS (37). Our study suggests that amelioration of the adiponectin signaling by augmenting the expression of its receptors after quercetin supplementation improves the metabolic features of PCOS. Importantly, it has been shown that quercetin supplementation in an animal model reduced insulin resistance and improved therapeutic effects in PCOS rats. Also, it was demonstrated that mechanistically quercetin inhibits the Toll-like receptor/NF-κB signaling pathway and in turn enhances the inflammatory microenvironment of the ovarian tissue of the PCOS rat model (39). Further evidence supports the anti-hypertensive and antiatherosclerotic advantages of quercitin supplementation in reducing atherosclerosis (24). Notably, quercetin supplementation improves dyslipidemia, hypertension and hyperinsulinemia as well as weight loss in diabetic mice, by reducing TNF-α production (40). Quercetin supplementation in animal models reduced glucose, insulin, triglyceride and cholesterol levels, however, it enhanced serum adiponectin levels (40, 41). Wein et al. (25) reported that the effect of quercetin in high fat-fed rats is due to the direct action of quercetin and independent of body stored fat size, improves insulin sensitivity and the HOMA index.

In parallel to the elevated expression of adiponectin receptors at the transcript level, quercetin also significantly enhanced the AMPK level. In this context, the positive correlation of *ADIPOR1* and *ADIPOR2* and serum AMPK overexpression suggests that quercetin supplementation exerts its beneficial metabolic effects in PCOS patients by targeting adiponectin and AMPK signaling pathways. While these three molecules are expressed in the ovary, it is reasonable to examine the biological actions of fuel sensors in fertility and it importance in fertility problems (2). We conclude that due to the detrimental inflammatory and metabolic characteristics of PCOS, quercetin, an antiinflammatory molecule, may mechanistically improve the metabolic features of PCOS by upregulating AMPK and adiponectin receptors. It would be worthy to confirm this finding by conducting in vitro studies on appropriate cell lines.
